# Muscular Activity and Fatigue in Lower-Limb and Trunk Muscles during Different Sit-To-Stand Tests

**DOI:** 10.1371/journal.pone.0141675

**Published:** 2015-10-27

**Authors:** Cristina Roldán-Jiménez, Paul Bennett, Antonio I. Cuesta-Vargas

**Affiliations:** 1 Department of Physiotherapy, Faculty of Health Sciences, Universidad de Malaga, Málaga, Spain; 2 School of Clinical Science, Faculty of Health Science, Queensland University Technology, Brisbane, Australia; University of Rome Foro Italico, ITALY

## Abstract

Sit-to-stand (STS) tests measure the ability to get up from a chair, reproducing an important component of daily living activity. As this functional task is essential for human independence, STS performance has been studied in the past decades using several methods, including electromyography. The aim of this study was to measure muscular activity and fatigue during different repetitions and speeds of STS tasks using surface electromyography in lower-limb and trunk muscles. This cross-sectional study recruited 30 healthy young adults. Average muscle activation, percentage of maximum voluntary contraction, muscle involvement in motion and fatigue were measured using surface electrodes placed on the medial gastrocnemius (MG), biceps femoris (BF), vastus medialis of the quadriceps (QM), the abdominal rectus (AR), erector spinae (ES), rectus femoris (RF), soleus (SO) and the tibialis anterior (TA). Five-repetition STS, 10-repetition STS and 30-second STS variants were performed. MG, BF, QM, ES and RF muscles showed differences in muscle activation, while QM, AR and ES muscles showed significant differences in MVC percentage. Also, significant differences in fatigue were found in QM muscle between different STS tests. There was no statistically significant fatigue in the BF, MG and SO muscles of the leg although there appeared to be a trend of increasing fatigue. These results could be useful in describing the functional movements of the STS test used in rehabilitation programs, notwithstanding that they were measured in healthy young subjects.

## Introduction

The use of the sit-to-stand (STS) test to measure muscle force was first described in 1985 in a study in which subjects had to rise from a chair ten times as quickly as possible, while the time required to perform the task was measured [1]. Over time there have been many variations in the test, including the number of times patients are required to get up from the chair.

The importance of STS is that getting up from a chair is a movement that is commonly repeated in activities of daily living and the results of this test provides a quantitative measure of an important functional activity [2]. It is one of the most important measures of physical ability and one of the most biomechanically demanding functional tasks, also being essential for patient independence [3,4]. For these reasons, studies using this test have been performed over several decades [5,6] and have been accomplished using various electronic devices, such as force platforms [7], optoelectronic systems [8], accelerometers [9–11], gyroscopes [12] and dynamometers [13]. This has allowed the analysis of the biomechanical characteristics of STS, for example the way it is affected by knee flexor and extensor muscle damage [14]; its variation depending on the position of the feet during the test [15]; or the relationship of the movement of the thoracic spine, lumbar spine and hip in the sagittal plane [16]. In 1990, a description of the elements of rising from a chair provided a quantitative characterization of the whole body movement [17]. In 2008, a normative database of STS was defined by quantitative kinematic and kinetic parameters, which also allowed comparing normal performance with impaired populations [18]. Besides defining normal parameters, STS has been used to discriminate between healthy subjects and those with impaired balance or vestibular disorder [19], as well as between young and old subjects and those at risk of falls [20,21].

In the field of electromyography (EMG) with reference to STS, there have been many different studies, for example: muscle activation in patients with knee arthroplasty [22]; the minimum peak joint moments of force required to perform the test [23]; and identification of which muscles can limit the performance of the activity because of their weakness [24]. Muscular demands have been measured both with and without the use of kinematic devices in the test [25]. Because the important role of strength during this task, EMG and kinematics has also been studied in older subjects during raising from a chair [26] and under different loaded conditions to induce muscle weakness [24,27]. Also, neuromuscular activity during the process of rising from a chair has been studied in different physical environments [28].

One of the variables most often considered in STS electromyographic studies is muscle fatigue, which is observed as a decrease in performance after exercise [29]. Motor control may be affected when fatigue is induced by the repetitive voluntary contractions of muscle groups used in the STS test [30]. In fact, there are studies that use the STS as a prior protocol to induce fatigue in the lower limbs [31].

STS has been well assessed in the literature, however, individual study designs and STS variants employed in each study makes comparison difficult. For example, speed has been proven to modify lower limbs joints movements and the centre of mass [32]. Therefore, it would be relevant to analyse how STS muscular activity is influenced by different speeds as well as increased number of repetitions in order to induce fatigue.

The aim of this study was to measure muscular activity and fatigue during different STS tasks and to analyse differences in those variables when STS repetitions and speed are increased, by using surface EMG in lower-limb and trunk muscles.

## Materials and Methods

### Subjects

This cross-sectional study recruited 30 healthy young adult subjects (18 men, 12 women) who met the inclusion criteria, and who were interested in taking part in the project. Students from the Faculty of Health Sciences (University of Málaga) were chosen.

Inclusion criteria were: aged between 18 and 35 years old; Body Mass Index (BMI) < 35; willing to join the study.

Subjects were excluded if they declined to participate in the study; suffered from a musculoskeletal, bone or joint injury; presented with any heart, pulmonary or mental illness; if they were taking medication; if they were not able to maintain minimum physical capabilities. Ethical approval for the study was granted by the Ethics Committee of the Faculty of Health Sciences, University of Malaga. The study complied with the principles laid out in the Declaration of Helsinki. Each participant was given an information sheet and provided written informed consent for participation. Participants were informed that participation was voluntary and they could withdraw at any point.

### Apparatus

Descriptive anthropometric independent variables were recorded. Dependent variables were obtained through electromyographic measures (Megawin 3.0.1., Mega Electronics Ltd, Kuopio, Finland) during the STS task. Rectification medium voltage (RMS averaging) was measured. The muscle activity of the medial gastrocnemius (MG), the biceps femoris (BF), the vastus medialis of the quadriceps (QM), the abdominal rectus (AR), the erector spinae (ES), the rectus femoris (RF), the soleus (SO) and the tibialis anterior (TA) on the subject’s dominant side were recorded. Three surface electrodes (Lessa, Barcelona, Spain, 2.5 cm) were used. To ensure good adhesion, the skin was washed with alcohol and shaved very gently so that any significant amount of hair was removed, but not completely shaved to avoid injury and increased blood flow in the area.

### Procedure

The investigator explained the task in a clear and concise manner, so that the participant understood the task they were required to perform. The start and end of the task were marked by an audible signal.

The study participants performed a series of exercises in the Laboratory of Human Movement at the Faculty of Health Sciences (University of Málaga).

**Fig 1:**
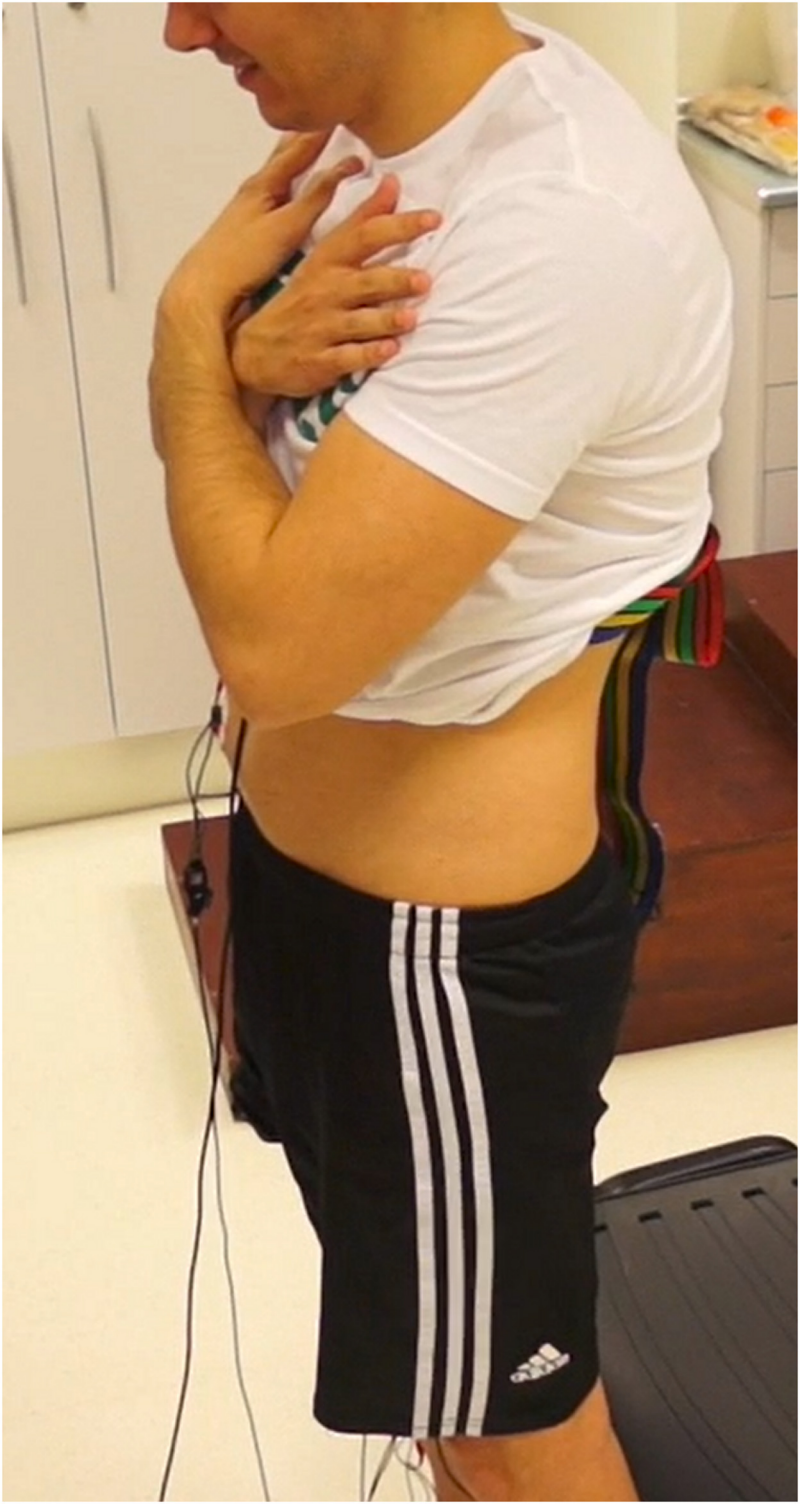
STS starting position.

The Maximal Voluntary Contraction test (MVC) was performed for each studied muscle according to Perotto [33]. Functional muscle testing for the MVC test was carried out according to Daniels [34], while electrode placement was carried out as described by Rainoldi [35]. Real activation (μV) was calculated from the maximum value minus the minimum value of this test to normalise the signals. Subsequently, variants of the sit-to-stand (STS) test were performed beginning in the standing position, with feet at the same distance apart as the hips, and upper limbs crossed in the anterior part, with bent elbows to avoid impulses (Fig 1). Given a signal from the investigator, the subject had to sit and rise from a 43 cm high chair at a speed of 40 beats per minute (bpm) provided by a digital metronome [Qwik Time QT-5 Metronome, China]. Variants of this test were:

- 5-STS: Sit down and stand up from the chair five times at a speed of 40 bpm.
- 10-STS: Sit down and stand up from the chair ten times at a speed of 40 bpm.
- 30-STS: Sit down and stand up from the chair as fast as possible for 30 seconds. The beginning and the end of the test were indicated by the investigator. The researcher observed throughout the test to ensure that the subject adjusted to the speed set by the metronome.

The dependent variables measured in the STS test were: average muscle activation (μV); percentage of MVC (%), normalised to represent the percentage of activation of each muscle relative to its real activation value recorded during the MVC test; muscle involvement in motion (%), representing the percentage distribution of electrical muscle activity; and fatigue, expressed in hertz (Hz) as median frequency (MF), which has shown to be a good parameter for assessing fatigue during dynamic conditions [36]

The reason for differentiating between normalised data (percentage of MVC, %) and muscle involvement (percentage distribution of electrical muscle activity, %) is that the EMG does not measure muscle power, but electrical activity released by the muscle, so that differences in the extent of activation measured may be due to the amount of muscle present and not to the amount of work being performed [37].

### Data analysis

For the analysis of the results a database was compiled from data collection notebooks and participant questionnaires. Following the intervention phase, descriptive statistics were calculated, with measures of central tendency and dispersion of the study variables. Student’s t-test was used to determine gender difference in anthropometric variables and real activation. ANOVA was performed for muscle activation, MVC percentage, muscle involvement and MF variables. SPSS 15.0 V Windows version was used. The values for muscle activity were recorded simultaneously using laptop hardware (Megawin 3.0.1.).

## Results

Anthropometric data from subjects are shown in Table 1.

**Table 1:** Descriptive and anthropometric variables in men and women (Mean ±SD).

Variables	Men (n = 18)	Women (n = 12)	P-value^a^
**Age (years)**	23.83±2.03	24±2.82	0.862
**Height (meters)**	1.69 ±0.42	1.65±0.05	0.000
**Weight (Kg)**	78.47±12.17	67.19±12.78	0.024
**BMI(Kg/m** ^**2**^ **)**	24.26±3.08	24.45±4.47	0.902

^**a**^ *p* values were calculated using t-student test.

During the MVC test, the minimum and maximum activation of each muscle was obtained, and real activation was calculated from these measures. The muscle with the highest real activation was QM in men and TA in women, while ES was the muscle with the least real activation in both men and women. In all muscles, higher values were found for males during the MVC test, and those differences were significant in MG, QM, ES and RF muscles (Table 2).

**Table 2:** Real activation (μV) obtained from MVC test (mean ± SD).

Muscles	Men (n = 18)	Women (n = 12)	P-value^a^
**Medial Gastrocnemius**	495.5±236.3	279.58±76.44	0.002
**Biceps Femoris**	630.5±260.36	595.58±258.29	0.721
**Vastus Medialis of Quadriceps**	985.61±455.91	476.91±196.71	0.000
**Abdominal Rectus**	761.94±363.03	484.83±395.53	0.065
**Erector Spinae**	527.11±245.52	232.63±232.21	0.004
**Rectus Femoris**	789.22±371.98	502.8±154.78	0.009
**Soleus**	635.27±366.31	608.83±315.8	0.835
**Tibialis Anterior**	976.55±389.02	860.75±82.80	0.352

^**a**^ *p* values were calculated using t-student test.

The average level of activation during each STS task was collated (Table 3). The highest activation was found in the QM muscle during 30-STS, while the lowest values were found in AR in all STS variants. Significant differences between different STS tasks were found in MG, BF, QM, ES and RF muscles.

**Table 3:** Muscle activation (μV) during STS test variants (mean ± SD) in total sample (n = 30) and ANOVA (f,p) test.

Muscle	5-STS	10-STS	30-STS	F^a^	P-value^a^
**Medial Gastrocnemius**	82.6±55.22	79.43±48.38	133.83±103.26	5.227	0.007
**Biceps Femoris**	110.50±66.76	115.10±79.47	191.17±160.53	5.055	0.008
**Vastus Medialis of Quadriceps**	238.73±92.37	238.53±99.8	331.40±153.06	6.158	0.003
**Abdominal Rectus**	40.23±54.9	46.93±63.28	75.46±92.3	2.027	0.138
**Erector Spinae**	90.64±61.58	97.63±87.17	169.69±188.01	3.453	0.036
**Rectus Femoris**	198.17±95.68	190.90±107.89	295.39±105.65	9.108	0.000
**Soleus**	184.37±392.89	111.20±60.22	177.07±103.39	0.867	0.424
**Tibialis Anterior**	259.50±79.25	237.53±67.95	275.87±118.21	1.339	0.268

^a^ F and P values were calculated using ANOVA

The percentage of MVC increased with increasing STS repetitions, except in the MG, SO and TA muscles, in which 5-STS had a higher percentage value than 10-STS. In all cases, MVC percentage was higher in 30-STS than in 5-STS, except in the SO muscle. The highest percentages were found in ES, QM, RF and MG in the 30-STS variant. Significant differences were found between different STS tasks in QM, AR and ES muscles. (Table 4).

**Table 4:** Percentage MVC during STS test variants (mean ± SD) in total sample (n = 30) and ANOVA (f,p) test.

Muscle	5-STS	10-STS	30-STS	F^a^	P-value^a^
**Medial Gastrocnemius**	25.87±21.33	25.04 ±19.99	43.20±44.95	3.013	0.054
**Biceps Femoris**	21.77±18.09	22.49±20	37.26±35.66	3.001	0.55
**Vastus Medialis of Quadriceps**	37.41±18.35	36.51±16.37	49.10±20.05	14.051	0.000
**Abdominal Rectus**	9.06±13.21	10.46±15.97	28.02±92.3	3.722	0.028
**Erector Spinae**	37.64±55.162	44.56±58.55	70.85±110.12	5.337	0.007
**Rectus Femoris**	30.55±20.540	33.61±25.65	46.15±27.23	1.444	0.242
**Soleus**	36.48±70.964	23.52±17.59	35.21±22.42	0.030	0.970
**Tibialis Anterior**	29.12±7.571	27.04±7.86	30.12±8.70	3.338	0.071

^a^ F and P values were calculated using ANOVA

**Fig 2:**
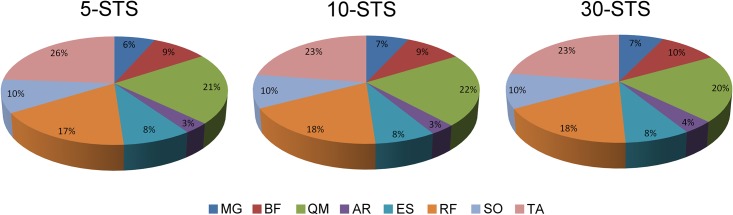
Distribution of muscle involvement in 5-STS, 10-STS and 30-STS.

Regarding the distribution of muscle involvement in the different STS tasks, as measured by the activation percentage of each muscle with respect to the total muscular activity signal recorded during the STS motion, the highest percentage was found in TA, followed by QM, RF and SO for all STS variants. More details are shown in Fig 2. In all muscles, no significant differences were found between different STS tasks (p>0.05).

**Fig 3:**
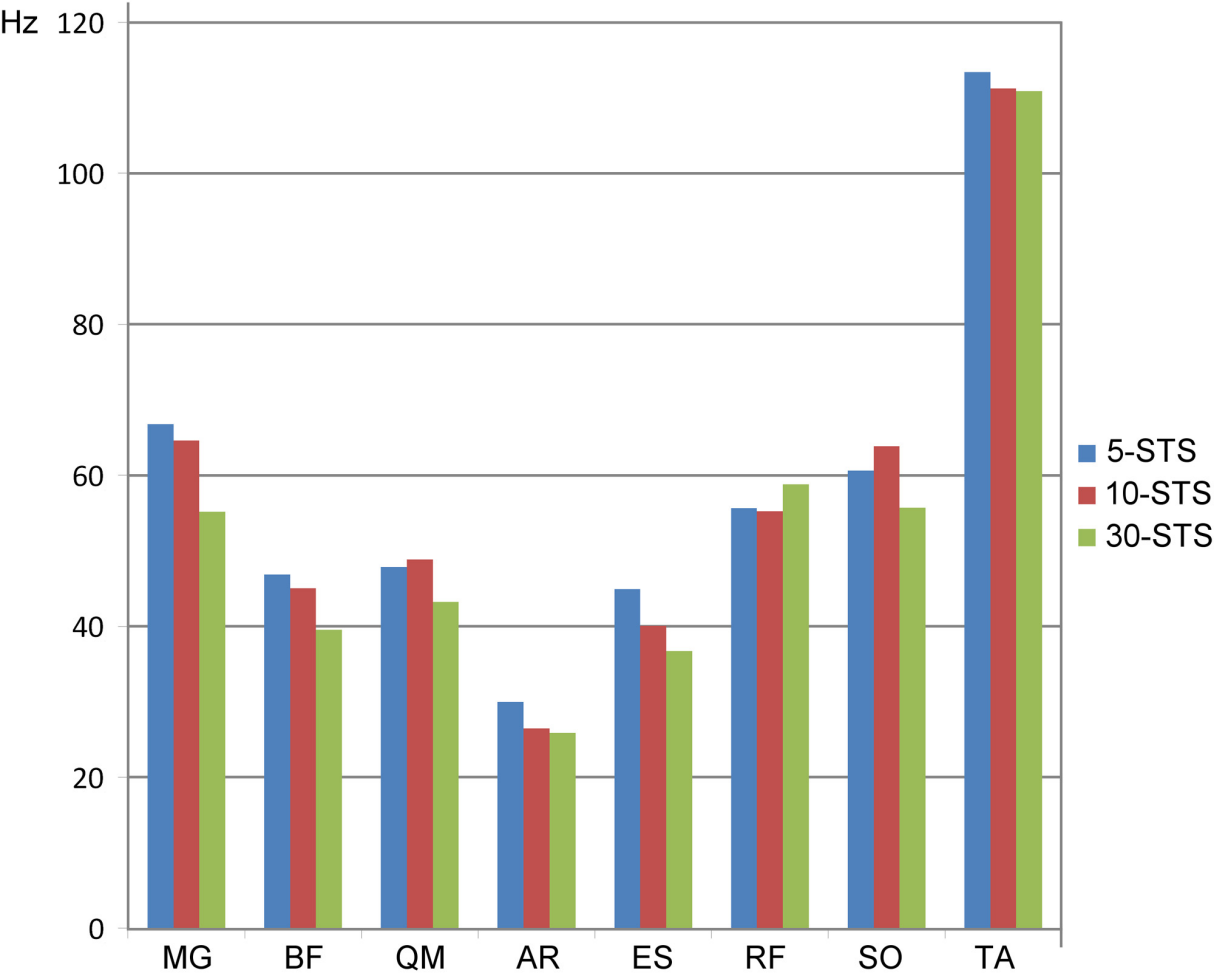
MF average for eight muscles in each STS task (Hz).

Regarding fatigue, MF varied in each muscle depending on the STS task performed. In MG, BF, AR, ES and TA muscles MF decreased with increasing STS repetitions. In QM and SO mean values were higher in 10-STS than 5-STS (Fig 3).

ANOVA showed that differences in MF were only significant for the QM muscle (p < 0.05). Also, the F value was higher than one in most cases, showing a trend in BF, MG, SO and ES (Table 5).

**Table 5:** ANOVA test for the MF variable.

Muscles	F	P-value
**Medial Gastrocnemius (MG)**	1.796	0.172
**Biceps Femoris (BF)**	2.530	0.086
**Vastus Medialis of Quadriceps (QM)**	3.781	0.027
**Abdominal Rectus (AR)**	0.499	0.609
**Erector Spinae (ES)**	1.170	0.316
**Rectus Femoris (RF)**	0.414	0.662
**Soleus (SO)**	1.559	0.216
**Tibialis Anterior (TA)**	0.032	0.968

## Discussion

The aim of this study was to analyse muscular activity and fatigue in different STS test repetition and speed variants using surface electromyography in lower-limb and trunk muscles.

Regarding muscle activation, significant differences were found between STS task in MG, BF and ES, in which activation increased when increasing repetitions. In QM and RF muscles differences where significant, but levels of activation were similar during 5-STS and 10-STS (speed at 40 bpm), and it increased with 30-STS. Concerning MVC percentage, QM, AR and ES muscles showed significant differences. Percentage in these muscles increased with the number of repetitions of each task. Therefore, these muscles are expected to achieve higher activation percentages when the number of STS repetitions increases. Regarding fatigue, the main finding was that there were significant differences in fatigue (expressed as MF), in QM muscle among the 5-STS, 10-STS and 30-STS variants. These data suggest that increased repetitions of the STS mainly affected the QM. This is in accordance with previous studies that suggests knee extensors are excessively loaded when muscle weakness develops under STS loaded condition [24]. There was also a trend of increasing fatigue in the BF, MG, SO and ES muscles of the leg with increasing repetitions in the 5-STS, 10-STS and 30-STS variants, although the differences were not statistically significant at p < 0.05. Therefore, it may be deducted from results that QM plays a main role in the execution of STS, as it is the muscle that presents differences in muscular activation, MVC percentage and MF between 5-STS, 10-STS and 30-STS tasks. Furthermore, preceded by TA, is the second muscle with a higher level of participation in muscle involvement in all tasks. Its importance is followed by ES, which also showed significant differences in muscle activation and MVC percentage. The importance of QM and ES is in line with a 1999 study which analysed STS transition, concluding that the knee extensors and flexors and the lumbar extensor muscles are the main executors of the task, while muscles such as the TA, SO and AR are used mainly in preparation for motion and for maintaining posture [38]. TA muscle has been defined as one of the first muscle activated during STS, as it anticipates postural adjustment aiming at stabilizing the foot before body mass forward [26]. This function could explain why TA showed the highest percentage of muscle involvement in this study. Along with SO muscle, they demonstrate constant muscular activity when load conditions during STS are modified [27]. In the present study, muscle activation and MVC percentages from these muscles did not present differences when modifying repetitions and speed neither. However, BF, RF, QM, MG activity increased which each load increment [27], which is in line with muscle activation increment when increasing speed and repetitions in this study.

Previous studies have studied MVC percentages during 5-STS. The same muscles as those examined here were studied in water and dry land during this task at a rate of 20 bpm. In dry land, the highest percentage MVC was found in QM, TA, RF and ES, ranging between 10% and 20% [28]. However, in this study, the highest percentage MVC was found in ES, QM, SO and RF, with a mean percentage value higher than 30%. Recently, 5-STS was studied with different pelvic sitting position at a self-selected speed. When it was performed with neutral pelvic position, percentage of MVC in QM muscle was 79.5±61.6 for men and 61.5±42.3 for women. With anterior pelvis tilt, percentages were 59.6±35.2 for men and 49.5±33.9 for women, showing significantly increased activation in the neutral pelvic position [39]. In the present study, values obtained in QM were lower, obtaining 37.41±18.35 in both genders. 5-STS task has also been compared with neutral position and hip abduction in older and young females. RF percentage of MVC was 34.11 ± 14.61 when it was performed naturally, and 34.09 ± 14.06 with hip abduction, with no significant differences between positions [40]. In the present study, MVC percentage during this task was 21.77±18.09 in both genders. 5-STS was also compared in healthy subjects and those who had suffered from stroke in different foot positions. Subjects were told to remain standing for 3 seconds and to sit down again at a comfortable speed. When performing this task in healthy subjects with a symmetric position, ES activation was found to be 156.9±75.8 μV [41]. However, in the present study, ES activation was 90.64±61.58. Those MVC percentage differences between studies could be due to muscular capacity of each individual, which could present a high variability in young people, as well as varied speed during the task, which has previously proven to influence parameters such as peak joint moment, phase changes or displacement [32].

Regarding muscle involvement, during getting up and sitting on a chair gesture has been compared between healthy subjects and those suffering intellectual disability. When performing that task in a 43 cm high chair at a speed 10 bpm, muscle involvement was 30% in TA, 19% in QM, 12% in ES, 11% in RF, 10% in SO, 7% in GM, 6% in BF and 5% in RA [42] in healthy subjects. In the present study, despite speed differences, results were similar: although percentage varied along different task (with no significant differences) the highest percentages were also found in TA (26–23%), QM (22–20%), RF (17–18%) and ES (8%) muscles. The great involvement of these muscles may be due to the fact that QM, RF and ES are considered as muscles fully involved in performing the STS [26]. Regarding TA, its function as foot stabilizer during this task has been previously explained in the text.

As it can be seen in the literature, there are a lot of STS studies focusing on different populations like elderly [29], fallers [10,43], subjects suffering from vestibular disorders or vestibular capacity [19], those who have suffered from stroke [44] or Parkinson disease [45]. However, those studies employ different methodology, like chair height, repetitions or speed. There are also investigations using STS test as a tool to asses fitness measure. However, they mainly focus on number of repetitions or the time to execute the action [46–48]. This study describes muscular activity and fatigue during 5 and 10 repetitions STS performed at a pre-set speed of 40 bpm, as well as 30 repetitions STS as fast as possible, to stablish references values in healthy subjects and analyse how electromyographic variables vary when STS features are modified. Quantifying gesture properties in healthy subjects may help clinicians to differentiate between different populations along STS variants. The results presented in this study could be useful in describing the functional movement of the STS in rehabilitation programs, considering that muscle activation and MVC percentage of some muscles participating in the motion oscillates depending on the repetitions performed, and that QM is the muscle most likely to become fatigued.

The findings of the present study should be taken with caution because the STS task has many variable factors that may influence the results, such as the chair seat height, the use of armrests [32] and variations in age [49] and BMI [50] of the subjects.

There are several limitations of this study that should be taken into account. The study did not employ any kinematic measurements. These measurements would detect variations in velocity or joint range during performance of the task between the 40 bpm variants (5-STS and 10-STS) and the 30-STS where sit-to-stand was performed as fast as possible for 30 seconds. Another limitation is that the results apply only to healthy young subjects and may not be generalisable to other populations. Future research is needed to investigate STS fatigue related to kinematic data. The use of more varied samples would allow the results to be more relevant to a heterogeneous population.

## Conclusions

This study describes muscular activity including average activation, MVC percentage, activity distribution and fatigue during different speed and repetitions of the STS task in healthy subjects. MG, BF, QM, ES and RF muscles showed differences in muscle activation, while QM, AR and ES muscles showed significant differences in MVC percentage. Also, significant differences in fatigue were found in QM muscle between different STS tests. These findings should be taken in consideration, as rising from a chair is a daily living activity task and widely used in rehabilitation.

## Supporting Information

S1 Supporting Information Dataset of study. (SAV)
